# A Hough-Space-Based Automatic Online Calibration Method for a Side-Rear-View Monitoring System

**DOI:** 10.3390/s20123407

**Published:** 2020-06-16

**Authors:** Jung Hyun Lee, Dong-Wook Lee

**Affiliations:** Department of Electronics and Electrical Engineering, Dongguk University, Seoul 04620, Korea; jhlee36@dongguk.edu

**Keywords:** side-rear-view monitoring system, automatic online calibration, Hough-space

## Abstract

We propose an automatic camera calibration method for a side-rear-view monitoring system in natural driving environments. The proposed method assumes that the camera is always located near the surface of the vehicle so that it always shoots a part of the vehicle. This method utilizes photographed vehicle information because the captured vehicle always appears stationary in the image, regardless of the surrounding environment. The proposed algorithm detects the vehicle from the image and computes the similarity score between the detected vehicle and the previously stored vehicle model. Conventional online calibration methods use additional equipment or operate only in specific driving environments. On the contrary, the proposed method is advantageous because it can automatically calibrate camera-based monitoring systems in any driving environment without using additional equipment. The calibration range of the automatic calibration method was verified through simulations and evaluated both quantitatively and qualitatively through actual driving experiments.

## 1. Introduction

In recent years, vision-based Advanced Driver Assistance Systems (ADAS) based on cameras have been developed continuously to provide safety and convenience to motorists. Vision-based ADAS employ the intrinsic and extrinsic parameters of cameras to provide a specific Field Of View (FOV). A Surround View Monitoring System, one of the vision-based ADAS, uses camera parameters to generate a bird’s eye view image with a FOV that contains all of the information around the vehicle [1,2]. A panoramic rear-view system also uses camera parameters to stitch side-view image and rear-view image to generate a panoramic image [3,4]. These vision-based ADAS transforms the captured image using camera parameters to generate the desired FOV image.

A side-rear-view monitoring system should especially provide a reliable FOV so that the driver can glean adequate information, and most countries legally specify the reliability of FOV. However, even though the same model vehicles are equipped with the same side-rear-view monitoring system devices, each monitoring system provides a different FOV due to manufacturing tolerances. FOV also changes when the same monitoring system device is mounted on different vehicles. Therefore, a side-rear-view monitoring system has to calibrate camera to provide uniform FOV even when various factors change. To provide consistent FOV according to the laws and circumstances of each country, control over the intrinsic and extrinsic parameters of cameras is required.

Camera calibration is one of the most useful methods for estimating the intrinsic and extrinsic parameters of a camera [5,6,7,8,9,10,11]. Camera calibration can improve camera performance by overcoming manufacturing tolerance limitations. Looser manufacturing tolerances can allow for lower cost and higher yield. Additionally, estimated parameters by calibration can be used to transform pixel-based metrics to physically based ones. This geometrical transformation enables FOV control.

Camera calibration for ADAS can be categorized into offline, self, and online calibration. Offline calibration methods use photographed targets, such as checker-patterns on a floor or wall [12,13,14,15,16,17,18]. This method is inconvenient because the size and position of the targets should be regulated depending on the location, orientation, and FOV of the camera. To this end, automobile manufacturers need to secure specialized facilities. Online calibration does not use specific targets and requires a moving camera. Traditional online calibration methods employ additional devices, such as encoders, Light Detection and Ranging (LiDAR) systems, odometry devices, and Inertial Measurement Units (IMU) [19,20,21,22] to overcome absence of specific targets. Other online calibration methods called self calibration [23,24,25] do not use additional devices but requires specific information about the road surface, such as lane markers [26,27,28,29]. However, it is not always possible to obtain specific information in the natural driving environment. In addition, self calibration methods have a constraint that some camera parameters must be known. Therefore, offline calibration must precede because the vehicle will not be operating on roads with lanes before it is sold. Unquestionably, the primary purpose of traditional online calibration is recalibration.

We propose a side-rear-view camera calibration method that is possible even if we do not know any camera parameters. Therefore, it does not require an offline calibration preprocessing step. In this method, a vision-based ADAS camera mounted near the side surface of a vehicle constantly photographs the vehicle. We call the part of the captured vehicle “Reflected-Vehicle Area (RVA)”, and we can extract the RVA not influenced by the driving environment. Therefore, the RVA is an essential prerequisite for our method.

A segmentation method with artificial intelligence such as deep learning is one of good solution to detect the RVA [30]. However, deep learning requires a huge amount of data based on the type of vehicle, camera parameters, and various driving environments. Collecting these data is very inconvenient and difficult. In order to overcome this inconvenience, we utilize widely known and uncomplicated image processing techniques to detect the RVA.

The proposed method detects the boundary of a reflected vehicle and computes the interior of the boundary as the RVA. The boundary of the reflected vehicle can be represented by any curve in the captured image. Random Sample Consensus (RANSAC) is a useful curve-fitting method. However, RANSAC is not always able to identify the optimal curve from moderately contaminated data [31]. Therefore, we eliminate contaminated data to the extent possible before utilizing RANSAC.

After the RVA is detected, the proposed method computes a similarity score between the detected RVA and a stored vehicle model. The similarity score can be calculated by the reprojection error minimization. To minimize reprojection error, we have to extract and match the features from the captured image and the stored image using Scale-Invariant Feature Transform (SIFT) or Speeded Up Robust Features (SURF) [32]. However, it is difficult to extract adequate numbers of feature points from two-dimensional vehicle images, which are required to apply image matching. The challenge is mainly due to the fact that the feature point is a corner where two straight lines with different slopes meet whereas RVA consists mostly of smooth curves. Image-template-matching methods can compute the similarity score without requiring feature point extraction. The template size should be small to facilitate the utilization of ring projection in conventional template-matching methods [33,34], but the RVA is too large from the viewpoint of applying ring projection. To solve this problem, Yang et al. studied large-scale rotation-invariant template matching [35]. This method uses color information, but the color of the RVA changes continuously because it reflects the surrounding environment. We propose a large-scale rotation-invariant template-matching method that computes the similarity score by using edge information instead of color information. The proposed algorithm utilizes the normalized 2D cross-correlation and the Hough space expressed in the Hesse normal form.

The rest of this paper is organized as follows: Section 2 reviews related works, and Section 3 describes the essential procedures of the proposed method. Section 4 presents simulation and experimental results. Finally, we conclude with a summary of the work in Section 5.

## 2. Related Works

This literature review focuses only on how to calibrate the parameters of a vehicle camera since camera calibration has been extensively researched for a long time in a wide range of fields. The previous methods can be classified as offline and online calibration according to which features are used. Additionally, online calibration can be categorized according to whether additional devices are used.

### 2.1. Offline Calibration

Offline calibration methods estimate camera parameters using special patterns consisting of edges, circles, or lines. Since these methods use precisely drawn patterns aligned with a camera-mounted vehicle, it is possible to accurately estimate the camera parameters. However, accurate calibration cannot be performed without special facilities aligning a vehicle and patterns in the precise location.

The A&G company [13] provides calibration facilities that can align the vehicle and the calibration patterns for highly accurate camera calibration. Xia et al. [14] used multiple patterns and cameras by minimizing the reprojection error. Mazzei et al. [15] also minimized the reprojection error of checkboard patterns’ corner locations to calibrate extrinsic parameters of the front view camera. Hold et al. [16] used a similar method using circle patterns on the ground. This method minimized the reprojection error of the centers of the circles. Tan et al. [17] drew an H-shaped pattern that consisted of two parallel lines and one perpendicular line to the vehicle. Li et al. [18] also used an H-shaped pattern to calibrate a rear-view camera.

### 2.2. Online Calibration with Additional Devices

Online calibration can estimate camera parameters using various sensors to utilize natural features instead of artificial patterns while driving. However, in terms of side-rear-view monitoring system calibration, this method has several drawbacks. Since the side-rear-view camera is looking at the horizon behind a vehicle rather than the road surface and part of the captured image is obscured by the driver’s vehicle, it is difficult to detect enough natural features. Therefore, feature-based algorithms are inappropriate for calibration of a side-rear-view monitoring system.

Wang et al. [19] proposed a camera-encoder system to estimate extrinsic parameters. They obtained the distance that camera traveled through the encoder and calculated the Euclidean distance between matched image feature points using feature extracting and matching algorithms. This method can estimate extrinsic parameters by comparing the Euclidean distance of matched image feature points with the camera movement distance. Schneider et al. [20] also utilized odometry, camera, and matched feature points for estimating intrinsic parameters. Chien et al. [21] used visual odometry and LiDAR for online calibration. Visual odometry determines equivalent odometry information using feature extracting and matching algorithms. Li et al. [22] utilized IMU to calibrate the camera. The data measured by the IMU is fed into a processor, which calculates the position.

### 2.3. Online Calibration without Additional Devices

Online calibration without additional devices extracts and matches natural feature points in image sequences. Since there is no other assistant equipment, these methods highly depend on feature extracting and matching algorithms. All of the introduced papers in this section utilized the road lanes as the feature points.

Xu et al. [26] and de Paula et al. [27] utilized the detection of two symmetrical lanes to calibrate cameras of the lane departure warning systems and augmented reality systems, respectively. Wang et al. [28] detected two symmetrical dotted lanes for online calibration. However, a side-rear-view camera captures few or no symmetrical lanes. Choi et al. [29] proposed the recalibration method for around view monitoring systems. This method can calibrate only when the road lanes around the vehicle are detected. However, road lanes near the vehicle are not captured by the side-rear-view camera.

## 3. Automatic Online Calibration

Automatic online calibration is a method that automatically calibrates the camera’s orientation and location in natural driving environments. However, the calibration method cannot change the orientation of a fixed camera in monitoring systems, which leads to deformed images. Therefore, we have to convert camera parameters into image deforming parameters.

The camera parameters can be classified into intrinsic and extrinsic parameters. Intrinsic parameters describe the optical properties of the camera, and extrinsic parameters describe the orientation and location of the camera. Since the optical properties of the manufactured camera such as the image sensor size, image sensor resolution, and distance between image sensor and lens hardly change, we assume that intrinsic parameters are constant. Therefore, we assume that intrinsic parameters are constant. Online calibration focuses only on the camera orientation because the orientation has considerably more influence on the image than the camera position [29,36]. Therefore, we also exclude camera location parameters, which is one of extrinsic parameters, from variables as well.

**Figure 1:**
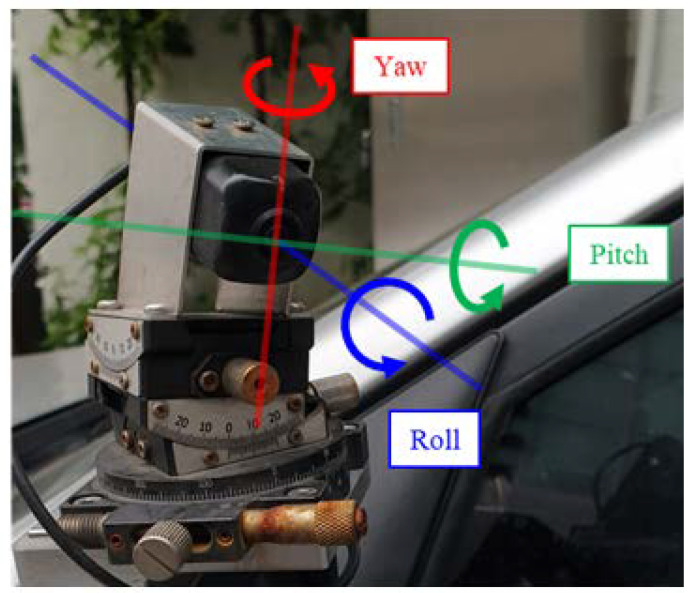
Parameterization of camera orientation.

The camera orientation can be expressed in terms of its roll, pitch, and yaw angles, as shown in Figure 1. When the camera rotates in the roll direction, the subject rotates in the image. When the camera rotates in the yaw and pitch directions, the subject moves in the horizontal and vertical directions, respectively, in the image. Therefore, the roll direction corresponds to image rotation, while the pitch and yaw directions correspond to image translation. By using this relationship, we can express camera orientation as image deforming parameters: image rotation and image translation parameters.

**Figure 2:**
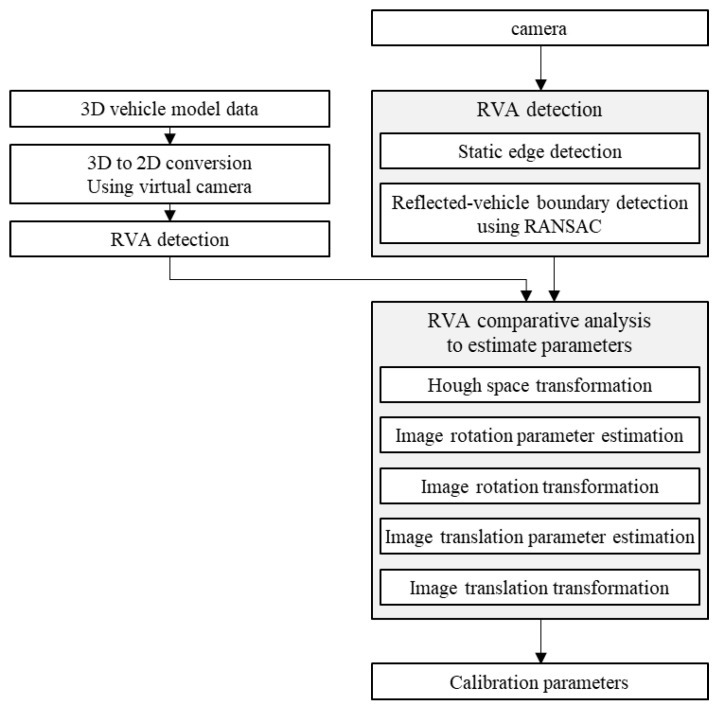
Block diagram of automatic online calibration.

We compare and analyze RVA of a pre-uploaded 3D vehicle model and RVA of a captured image in order to estimate the parameters. An RVA detection step has to be preceded for comparative analysis. RVA data in the image space is converted into the Hough space to estimate the image rotation parameters. We can estimate the image translation parameters using two RVA data with no image rotation difference. Figure 2 shows the procedure of the proposed automatic online calibration.

### 3.1. Reflected-Vehicle Area Detection

RVA is a part of the driver’s vehicle photographed in the image. The algorithm for detecting RVA consists of two steps. The first step involves preprocessing to improve the accuracy of the second step and to eliminate, to the extent possible, the data that are not related to the reflected vehicle. In the second step, we utilized RANSAC to find the reflected-vehicle boundary and determine the inside of this boundary as the RVA. RANSAC is an iterative curve-fitting method for estimating the parameters of a mathematical model and classifying data into inliers and outliers. Inliers are the data whose distribution can be explained by some set of model parameters, and outliers are the data that do not fit the model. Therefore, outliers do not influence the estimated parameters. For this reason, RANSAC is used for outlier detection as well [37].

**Figure 3:**
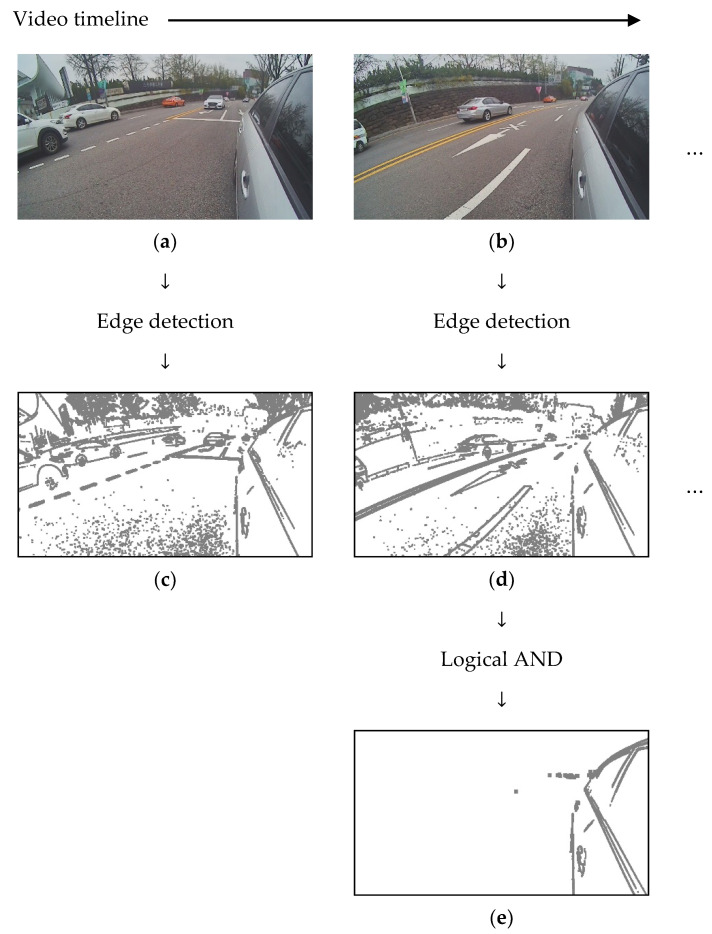
Concept of static edge detection. (**a**) First captured image; (**b**) second captured image; (**c**) edge image of (**a**); (**d**) edge image of (**b**); and (**e**) static edge image after logical and processing.

In the first step, we eliminate outliers to improve the accuracy of RANSAC, which is inversely proportional to the number of outliers. We assume that the edge points of the reflected vehicle are inliers and all other points are outliers. The edge points of RVA always appear stationary because the moving speed and direction of the camera installed on the vehicle are the same as those of the vehicle. Therefore, we detected edges that do not change over time, and we call this process “static edge detection”. To detect static edge points, we capture multiple images over a certain time period and detect the edges of each captured image using the Sobel filter. Thereafter, we could detect static edges by applying the logical and operation to each pixel coordinate. Figure 3 shows an example of the detection of static edges.

**Figure 4:**
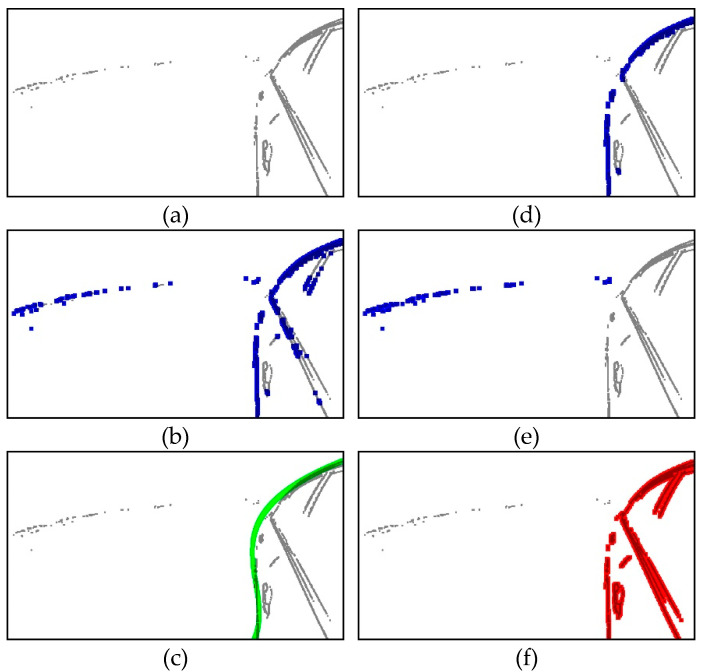
Example of reflected-vehicle area detection using Random Sample Consensus (RANSAC). (**a**) Static edge image; (**b**) determined candidates of the reflected-vehicle boundary; (**c**) estimated reflected-vehicle boundary using RANSAC; (**d**) identified reflected-vehicle boundary points from (**b**); (**e**) eliminated static edge points outside Reflected-Vehicle Area (RVA); and (**f**) static edge points inside RVA.

Edge detection using the Sobel filter does not guarantee robust results because it uses static parameters of filter to detect edges of dynamic images. However, the proposed static edge detection can overcome this problem by collecting lots of edge information from multiple images. Therefore, we must capture an adequate number of images to eliminate static edge points outside RVA to the extent possible. The static edge image in Figure 4a confirms that the static edge points inside RVA also form a curve as distinct as the reflected-vehicle boundary. Therefore, if there are several static edge points in each row of the image, only the leftmost static edge point is set as the candidate of the reflected-vehicle boundary. This process not only eliminates the static edge points inside the RVA but also represents candidates as a bijection function. Figure 4b shows the candidate points of the reflected-vehicle boundary. In this figure, most of the static edge points inside RVA are not candidates. After determining the candidate group, we utilize RANSAC to detect RVA in the second step.

**Figure 5:**
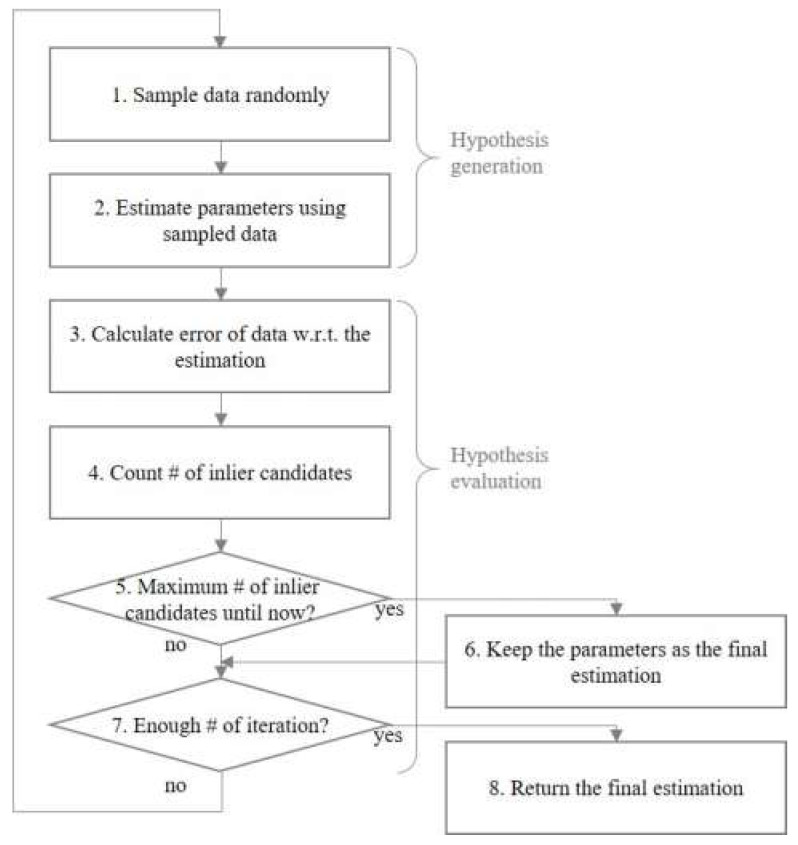
Flowchart of RANSAC.

In the first step, we eliminated most of the static edge points, except for the points of the reflected-vehicle boundary. We utilized RANSAC to categorize the candidates into the reflected-vehicle boundary (inliers) and the others (outliers) in the second step. RANSAC is an iterative method involving two phases: hypothesis generation and hypothesis evaluation, as shown in Figure 5.

RANSAC generates the hypothesis for line fitting by randomly sampling data and estimating parameters using the sampled data. The highest score of hypothesis evaluation is given when all randomly picked-up data are inliers. Therefore, hypothesis generation and evaluation must be iterated so that all randomly picked-up data are inliers.

The probability p that RANSAC will select all inlier samples at once is as follows.

(1) $$p=1-{\left(1-\gamma^{s}\right)}^{N}$$

where γ is the number of inliers divided by the number of points in the data, s is the number of samples selected each time, and N is the number of iterations. Equation (2) can be used to determine the number of iterations.

(2) $$N=\frac{\log\left(1-p\right)}{\log\left(1-\gamma^{s}\right)}$$

We can determine the variables γ and s experimentally, but the probability p can only be determined empirically. After hypothesis generation, RANSAC calculates the error that the distance datum has to the estimated line and counts the number of inliers within a predefined threshold to evaluate the hypothesis. To predefine the threshold, we assumed that the error follows a normal distribution. In statistics, the empirical rule is expressed as follows: X is an observation from a normally distributed random variable, μ is the mean of the distribution, and σ is its standard deviation.

(3) $$\begin{array}{c} \mathrm{Pr}\left(\mu -1\sigma \leq X\leq \mu +1\sigma \right)\approx 0.6827\\ \mathrm{Pr}\left(\mu -2\sigma \leq X\leq \mu +2\sigma \right)\approx 0.9545\\ \mathrm{Pr}\left(\mu -3\sigma \leq X\leq \mu +3\sigma \right)\approx 0.9973 \end{array}$$

We obtained the standard deviation of the inliers σ and then predefine the threshold between 2σ and 3σ so that RANSAC can select inliers with a probability of 95% or higher. Finally, we could detect the reflected-vehicle boundary by using RANSAC.

Boundary of a reflected-vehicle can be represented by a smooth curve. However, the boundary changes depend on the vehicle type and camera parameters. Therefore, we used a third-order equation as a model of RANSAC as shown below.

(4) $$f\left(v\right)=a_{0}+a_{1}v+a_{2}v^{2}+a_{3}v^{3}$$

where v is a horizontal direction coordinate of RVA point. Additionally, the order of the equation may increase as needed.

Figure 4c shows a reflected-vehicle boundary curve estimated using RANSAC. The blue points in Figure 4b are the candidates of a reflected-vehicle boundary, and these points are used as the input data for RANSAC. Figure 4c shows the curve with the most inliers, as estimated using RANSAC, and the blue points in Figure 4d are the candidates identified as inliers by RANSAC.

We assume that the interior of an estimated reflected-vehicle boundary is RVA. Figure 4e,f show the static edge points outside RVA and the static edge points inside RVA, respectively.

In the next section, we presented an automatic calibration method that employs these static edge points.

### 3.2. RVA Comparative Analysis to Estimate Parameters

**Figure 6:**
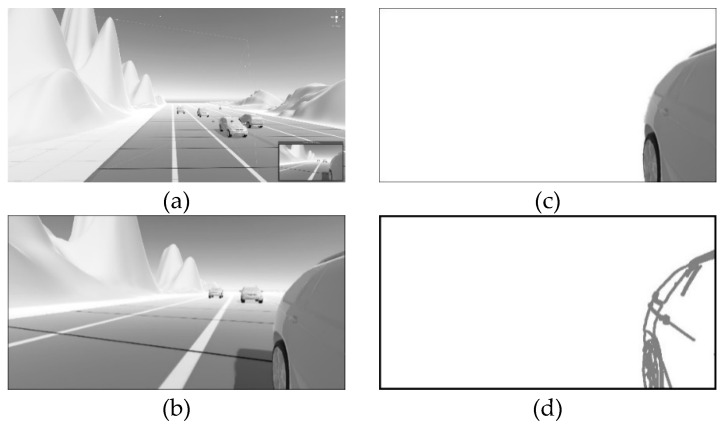
Process of converting a 3D vehicle model into an edge image by using the Unity program. (**a**) 3D virtual space of the Unity program; (**b**) photograph captured using a virtual camera; (**c**) reflected-vehicle area of image captured using a virtual camera; and (**d**) edge image of a 3D vehicle model in the reflected-vehicle area.

We estimated the image rotation and translation parameters that represent the camera orientation by comparing RVA with the stored vehicle model. The stored vehicle model must be converted into an edge image to compare it with the RVA consisting of static edge points. Vehicle manufacturers may provide three-dimensional (3D) vehicle model data; if that is not the case, we can construct the data by using a 3D scanner. Then, we can regulate the camera position, orientation, and FOV and shoot a 3D vehicle in 3D virtual space. The Unity program is useful for regulating the virtual camera and for clicking pictures with it in 3D virtual space [38]. We applied edge detection to images captured using the virtual camera to obtain a reflected-vehicle edge image of the stored vehicle model. Figure 6 shows the process of converting a 3D vehicle model into an edge image by using the Unity program.

After converting the edge image from the 3D vehicle model, we utilized the Hough space to compare the converted edge image of the 3D vehicle model and the result of reflected-vehicle area detection in Section 2. The Hough space is a set of values that transform the edge points of the RVA into the Hesse normal form [39]. Equation (5) represents the Hesse normal form.

(5) r=xcosθ+ysinθ

**Figure 7:**
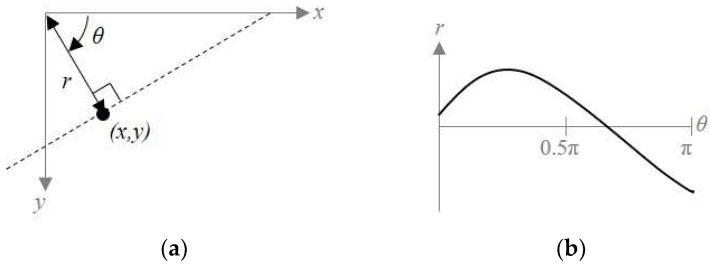
Parameterization of image space and Hough space. (**a**) A point in the image space, and (**b**) a curve corresponding to an image space point in the Hough space.

The coordinate (x,y) can be expressed as (r,θ) by using Equation (5), and we can visualize (r,θ) as a curve. Figure 7 shows a visualized curve corresponding to an image space point in the Hough space. We assumed that this curve can be expressed as r=h(θ).

**Figure 8:**
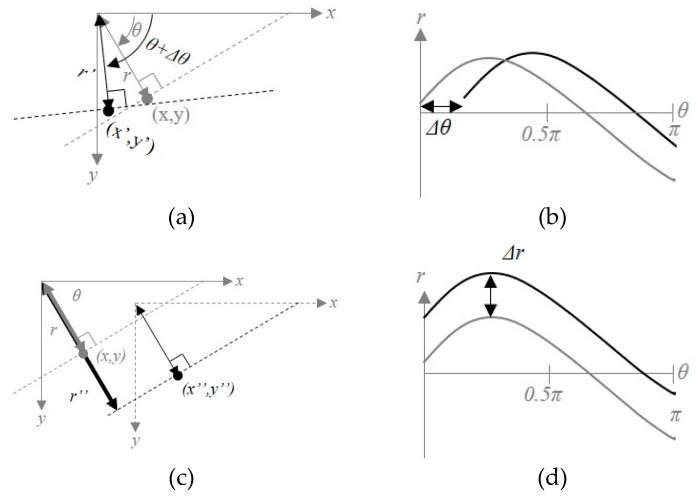
Parameterization of image space and Hough space by means of similarity transformation. (**a**) Rotation transformation in image space; (**b**) rotation transformation in Hough space; (**c**) translation transformation in image space; and (**d**) translation transformation in Hough space.

If the coordinate (x,y) is rotated by Δθ and moved to $\left(x^{\prime },y^{\prime }\right)$, a degree-shift of Δθ occurs in the Hough space, and if (Δx,Δy) image translation occurs, r-shifting occurs in the Hough space, as shown in Figure 8. This phenomenon indicates that the parameter θ and (Δx,Δy) image translation are independent of each other. Therefore, the Hesse normal form can be re-expressed by considering that image rotation and translation occur simultaneously:

(6) $$r+\Delta r=x^{\prime \prime }\cos\left(\theta +\Delta \theta \right)+y^{\prime \prime }\sin\left(\theta +\Delta \theta \right).$$

**Figure 9:**
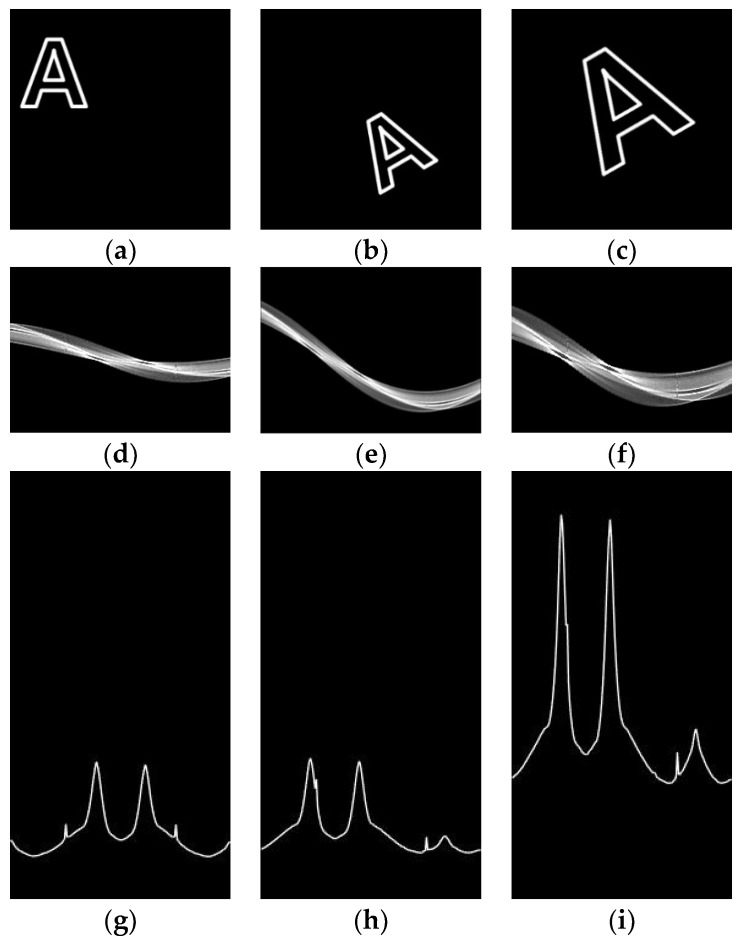
Example of Hough space data changed by image transformation. (**a**) A test image; (**b**) result of rotating and translating image (**a**); (**c**) result of scaling and translating image (**b**); (**d**) result of converting data (**a**) to the Hough space, where row is θ and column is r; (**e**) result of converting data (**b**); (**f**) result of converting data (**c**); (**g**) variance of (**d**) corresponding to θ, where row is θ, and column is variance v(θ); (**h**) variance of (**e**); and (**i**) variance of (**f**).

By using Equation (6), the Hough space curve r=h(θ) can be re-expressed as r+Δr=h(θ+Δθ). We can estimate rotational similarity by comparing the difference between h(θ) and h(θ+Δθ). r=h(θ) denotes a curve in the Hough space corresponding to one point in the image space. Many points exist in the image space, so we calculate the variance of h(θ) corresponding to each θ to solve this problem.

(7) $$v\left(\theta \right)=\frac{1}{N-1}\overset{N}{\underset{i=1}{\sum }}{\left|h_{i}\left(\theta \right)-\mu_{h}\right|}^{2}$$

where v(θ) is the variance of h(θ) corresponding to θ, N is the number of edge points, $h_{i}\left(\theta \right)$ is h(θ) corresponding to the ith edge point, and $\mu_{h}=\frac{1}{N}\sum_{i=1}^{N}h_{i}\left(\theta \right)$. Figure 9 shows an example of how (r,θ) of the Hough space and variance v(θ) are changed by image transformation. Figure 9g–i show that the variance v(θ) is shifted in the vertical direction owing to image rotation, and the amplitude of v(θ) is stretched in the horizontal direction owing to image scaling. Moreover, the effect of image translation is rarely seen in the Hough space. Therefore, we can estimate the rotational similarity between Figure 9a,b by computing the degree-shifting between variances v(θ), as shown in Figure 9g,h.

We utilized normalized cross-correlation to calculate the degree-shifting between $v_{m}\left(\theta \right)$ and $v_{c}\left(\theta \right)$, where $v_{m}\left(\theta \right)$ is a curve corresponding to the 3D vehicle model, and $v_{c}\left(\theta \right)$ is a curve corresponding to a static edge image of RVA. Normalization is applied to calculate the degree-shifting when the amplitude difference between two signals is large, as shown in Figure 9g,i. The normalized cross-correlation is one of the proper solutions for estimating the relationship between two signals, and it is expressed as follows:

(8) $$R\left(\phi \right)=\frac{1}{K}\sum_{k}\frac{{\left(v_{m}\left(\theta \right)\right)}^{*}v_{c}\left(\theta +\phi \right)}{\sigma_{v_{m}}\sigma_{v_{c}}},$$

where $\sigma_{v_{m}}$ is the variance of $v_{m}\left(\theta \right)$, $\sigma_{v_{c}}$ is the variance of $v_{c}\left(\theta \right)$, ∗ is the complex conjugate, and K is the length of valid signals. Then, we can obtain the rotational similarity Δθ by using Equation (9).

(9) $$\Delta \theta =\underset{\phi }{\mathrm{argmax}}\left(R\left(\phi \right)\right)$$

If the camera image is calibrated using the estimated rotational similarity score, only translational similarity remains to be determined. We can obtain translational similarity from the normalized 2D cross-correlation, which is widely used in computer vision [40].

(10) $$\gamma \left(u,v\right)=\frac{\sum_{x,y}\left[I_{m}\left(x,y\right)-\mu_{I_{m}}\right]\left[{\hat{I}}_{c}\left(x-u,x-v\right)-\mu_{{\hat{I}}_{c}}\right]}{{\left\{\sum_{x,y}{\left[I_{m}\left(x,y\right)-\mu_{I_{m}}\right]}^{2}\sum_{x,y}{\left[{\hat{I}}_{c}\left(x-u,x-v\right)-\mu_{{\hat{I}}_{c}}\right]}^{2}\right\}}^{0.5}}$$

where γ(u,v) denotes the normalized 2D cross-correlation data at (u,v), $I_{m}$ the edge image of the 3D vehicle model, ${\hat{I}}_{c}$ the rotation-corrected camera image, and $\mu_{I_{m}}$ and $\mu_{{\hat{I}}_{c}}$ the averages of $I_{m}$ and ${\hat{I}}_{c}$, respectively. Then, we can compute the translational similarity by using Equation (11).

(11) $$\left(\Delta x,\Delta y\right)=\underset{u,v}{\mathrm{argmax}}\left(\gamma \left(u,v\right)\right)$$

Finally, we can construct a similarity matrix from Δx, Δy, and Δθ, and calibrate the image captured by the camera as follows:

(12) $$H_{S}=\left[\begin{array}{cc} R & t\\ 0 & 1 \end{array}\right]=\left[\begin{array}{ccc} \cos\Delta \theta & -\sin\Delta \theta & \Delta x\\ \sin\Delta \theta & \cos\Delta \theta & \Delta y\\ 0 & 0 & 1 \end{array}\right],$$

where $H_{S}$ is the similarity matrix, R is the image rotation matrix, and t is the image translation matrix instead of 3D camera orientation.

## 4. Simulation and Experimental Results

**Figure 10:**
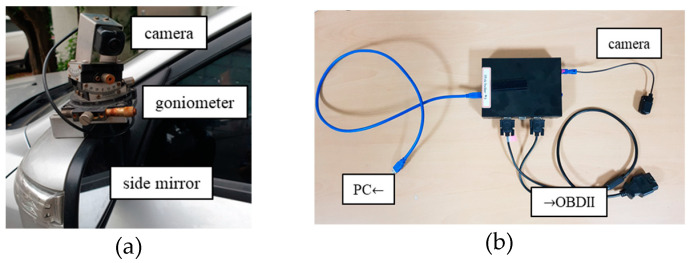
(**a**) Camera and goniometer used in the experiment and (**b**) grabber equipment for synchronizing and acquiring Controller Area Network (CAN) data and Low-Voltage Differential Signaling (LVDS) camera data.

We performed several simulations and experiments to illustrate the performance of the proposed method. The purpose of the first experiment was to determine the number of captured images for static edge detection. The second experiment was performed and repeated to validate the effect of the driving environment on the automatic calibration. We compared our method with previous methods in the third experiment. The final experiment indicated the constraints of the proposed method. For these experiments, we installed High-Definition Low-Voltage Differential Signaling (HD LVDS) cameras with 60-degree FOV and a resolution of 1280 px × 720 px on the vehicle’s left- and right-side mirror. These values were chosen due to their similarities to humans’ angle of view. The camera was equipped with a three-axis goniometer to change its orientation, as shown in Figure 10a. We also produced and installed grabber equipment to acquire Controller Area Network (CAN) data and LVDS camera images, as shown in Figure 10b. The proposed algorithm was implemented in C++ on a portable PC. We analyzed the CAN data via the car’ On-Board Diagnostic II (OBDII) port to detect vehicle speed and captured images only when the vehicle was in motion.

### 4.1. Experiments for Determining An Appropriate Number of Captured Images

**Figure 11:**
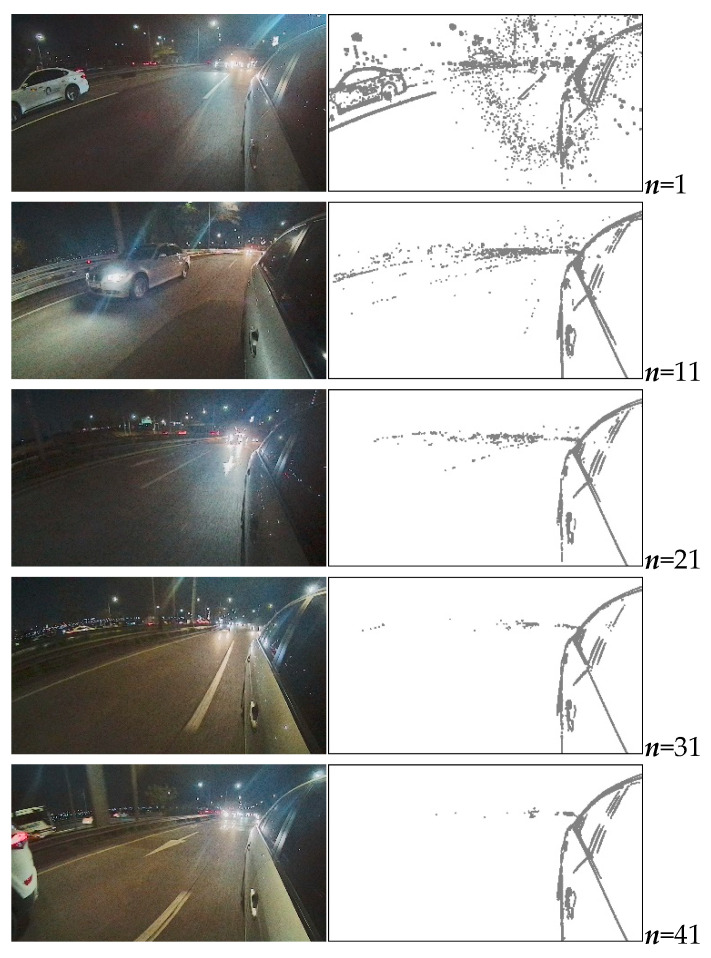
Left side: ***n***th captured image, where ***n*** is the number of images captured; right side: static edge points of reflected-vehicle edge detection according to ***n***.

We compared the results of reflected-vehicle edge detection by changing the number of captured images to determine the appropriate number of captured images required for the purpose. Figure 11 shows the results of reflected-vehicle edge detection as a function of the number of captured images. The static edge points outside RVA were eliminated as the number of captured images increased. However, as the number of captured images increased, the time and memory costs increased as well. Due to this tradeoff relationship, we repeated this experiment in different driving environments and generalized the relationship between the number of captured images and the number of static edge points outside RVA.

**Figure 12:**
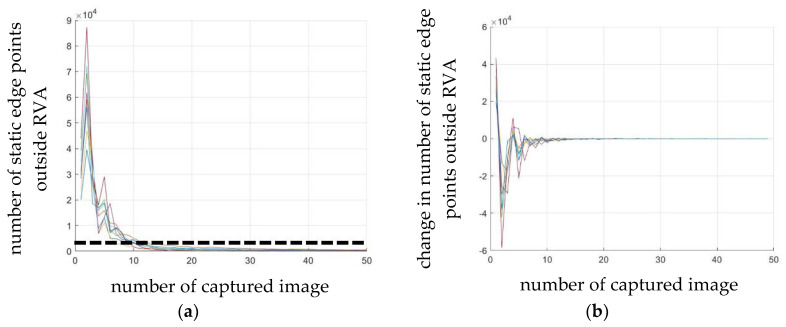
(**a**) Number of static edge points outside RVA according to the number of captured images, where the black dash line indicates that the ratio of the number of static edge points outside RVA to the number of static edge points is 0.5 and (**b**) change in the number of static edge points outside RVA according to the number of captured images.

Figure 12 shows the relationship between the number of static edge points outside RVA and the number of captured images. If more than 15 captured images were used, the ratio of the number of static edge points outside RVA to the number in the number of static edge points outside RVA converged to zero, as shown in Figure 12. Therefore, at least 15 captured images should be used so that the proportion of the number of static edge points outside RVA is less than 50%. Furthermore, we could eliminate a greater number of static edge points outside RVA by capturing more than 15 images, depending on the operating time and the computing power of the equipment.

### 4.2. Field Experiments for Quantitative and Qualitative Evaluation

**Figure 13:**
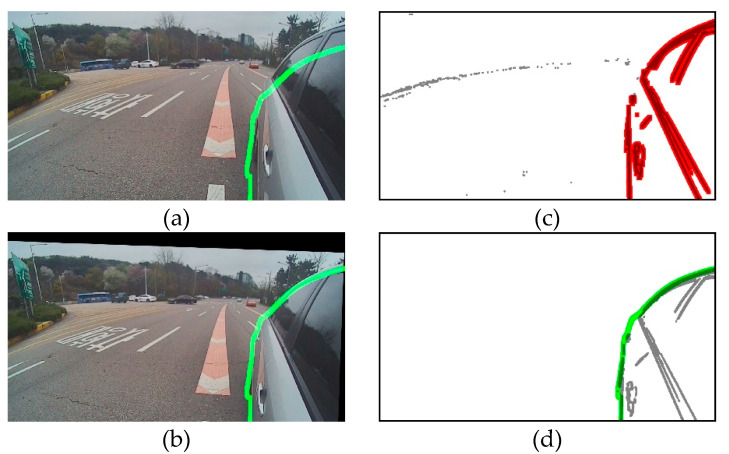
Field experiment result with speed limits of 80 km/h. (**a**) Captured image, where the green line denotes the boundary of the 3D vehicle model; (**b**) calibrated image, where the green line denotes the boundary of the 3D vehicle model; (**c**) RVA detection, where red points are static edge points inside RVA and gray points are static edge points outside RVA; and (**d**) edge image of 3D vehicle model, where the green line denotes the boundary of the 3D vehicle model.

We conducted experiments to verify each of the algorithms applied to the proposed method in natural driving environments. We used a goniometer and artificially regulated the camera orientation by 5° per axis. Figure 13 shows the process of the proposed method. Figure 13c shows the results of static edge detection and RVA detection. The static edge points outside RVA have been appropriately eliminated. Figure 13a shows one of the captured images, Figure 13b shows the result of automatic calibration of the captured images, and the green curves indicate the boundary of the 3D vehicle model. As shown in Figure 13b, the green curve almost matches the boundary of the reflected vehicle in the calibrated image. This result indicates that the proposed automatic calibration is apt for a side-rear-view monitoring system and that the proposed automatic calibration is accurate even when the camera orientation changes.

**Figure 14:**
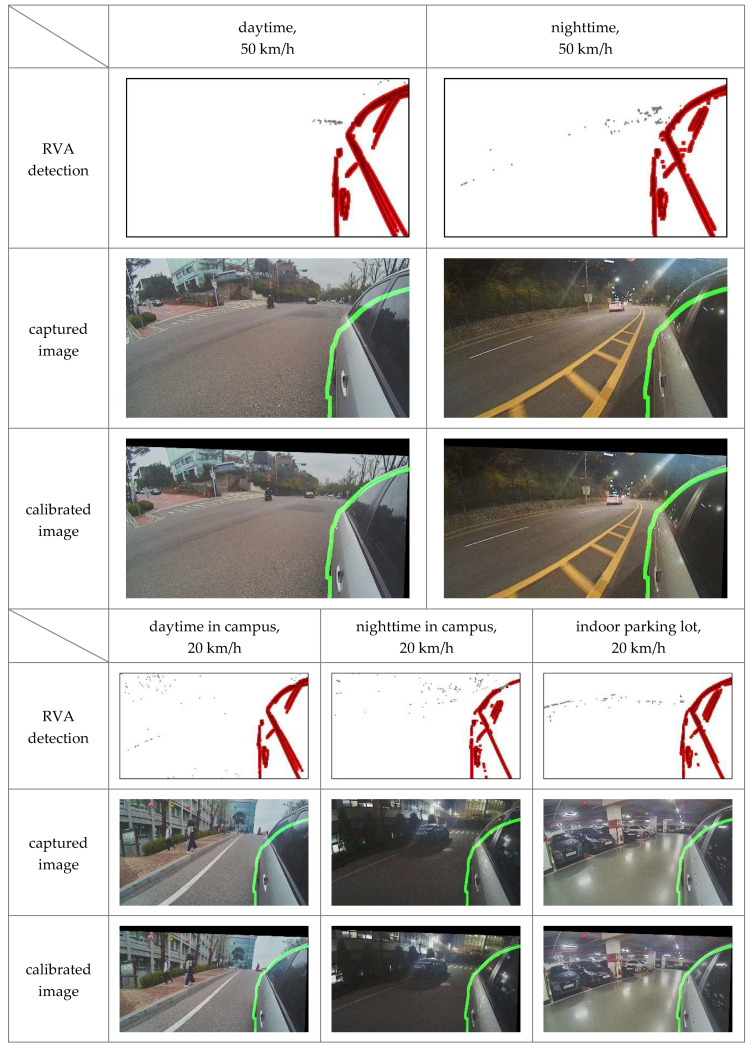
Results obtained using the proposed method in different experimental environments where the red points are the static edge points inside RVA, and the green line denotes the boundary of the 3D vehicle model.

We repeated the driving test in various environments without changing the camera orientation and the edge image of the 3D vehicle model to verify whether the proposed method can provide consistent results. We drove through a school campus with a speed limit of 20 km/h, city road with a speed limit of 50 km/h, speedway with a speed limit of 80 km/h, and an indoor parking lot during the day and night. Figure 14 shows the results of the experiment in each environment. The static edge points outside RVA appeared near the horizon when driving at the speed of 50 km/h or more. These static edges mostly indicate a horizontal vanishing line whose position hardly changed in the image. In the school campus or the underground parking lot, static edge points outside RVA were randomly scattered. Therefore, reflected-vehicle edge detection was essential when driving on a natural road with horizon views.

The static edge points inside RVA were more evident in the daytime than in the nighttime. Naturally, the edge of the car was more clearly visible in a bright environment than in a dark environment. Moreover, RVA detection results were evident in the dark when high-speed roads and parking lots were well lit. On the contrary, fewer static edge points inside RVA were detected in the campus during the night-time because of poorer lighting conditions compared to those in the other environments. Nevertheless, the proposed method took advantage of the Hough space (see Section 3) to ensure that the calibration can be performed even when there are only a few static edge points inside RVA.

We must know all camera parameters except rotation parameters to implement existing methods, whereas our method operates with unknown camera parameters. Moreover, the ground truths of the camera parameters installed in the vehicle were not available [19,41,42,43]. Therefore, we repeated this experiment 100 times and used precision, recall, and Root Mean Squared Error (RMSE) as the quantitative evaluation indexes. Furthermore, we experimented with a 150-degree FOV camera with lens distortion, a 115-degree FOV camera with lens distortion, and a 150-degree FOV camera without lens distortion to confirm the applicability of the algorithm to other ADAS cameras.

#### 4.2.1. Precision, Recall, and RMSE

Precision and recall can be obtained by calculating true positive (TP), false positive (FP), false negative (FN), and true negative (TN) [44].

(13) $$\begin{array}{c} \mathrm{precision}=\frac{\mathrm{TP}}{\mathrm{TP}+\mathrm{FP}}\\ \mathrm{recall}=\frac{\mathrm{TP}}{\mathrm{TP}+\mathrm{FN}} \end{array}$$

TP, FP, FN, and TN are defined in Table 1, where $I_{m}$ denotes the edge image of the 3D vehicle model, ${\hat{I}}_{c}$ is a calibrated image, $S_{m}$ is RVA of $I_{m}$, ${\left(S_{m}\right)}^{c}$ is area outside RVA of $I_{m}$, $S_{c}$ is RVA of ${\hat{I}}_{c}$, and ${\left(S_{c}\right)}^{c}$ is area outside RVA of ${\hat{I}}_{c}$.

**Figure 15:**
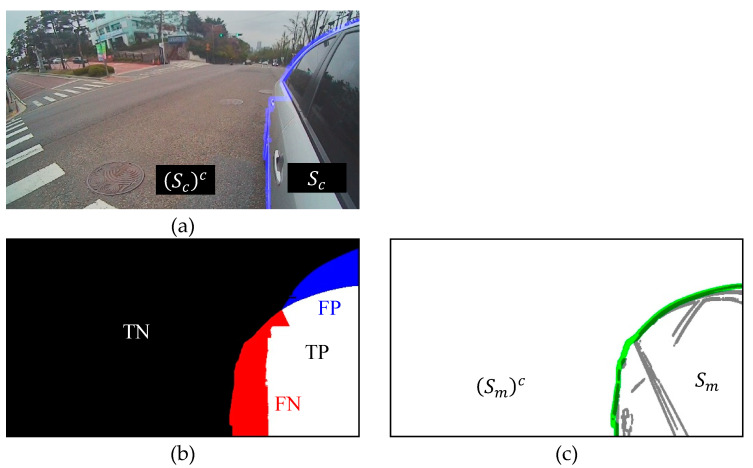
Visualization and parameterization of TP, FP, FN, and TN. (**a**) Captured image where the blue line is the boundary of RVA of captured image and $S_{c}$ is RVA of calibrated image; (**b**) TP $=S_{m}\cap S_{c}$, TN $={\left(S_{m}\right)}^{c}\cap {\left(S_{c}\right)}^{c}$, FN $=S_{m}\cap {\left(S_{c}\right)}^{c}$, and FP $={\left(S_{m}\right)}^{c}\cap S_{c}$; and (**c**) edge image of the 3D vehicle model where the green line is the boundary of 3D vehicle model and $S_{m}$ is the RVA of the edge image of the 3D vehicle model.

**Figure 16:**
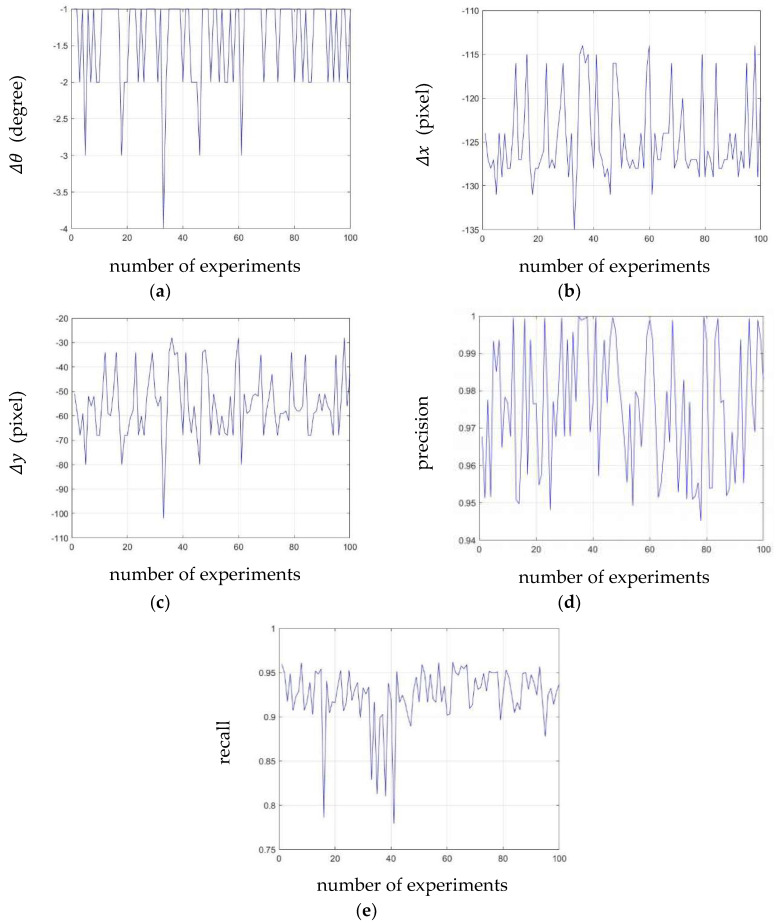
The result of 100 repeated experiments. (**a**) Estimated rotational similarity; (**b**) *x*-axis translational similarity; (**c**) *y*-axis translational similarity; (**d**) precision values; and (**e**) recall values.

Figure 15 shows the TP, FP, FN, and TN areas visually. TP is the number of intersection points between $S_{m}$ and $S_{c}$. The red region FN denotes the number of intersection points between $S_{m}$ and ${\left(S_{c}\right)}^{c}$. We could calculate FP and TN as well in the same manner. The RMSE is defined as follows.

(14) $$\begin{array}{c} RMSE_{rot}=\sqrt{\frac{1}{N}\sum_{n}{\left|\Delta \theta \left(n\right)-\mu_{\Delta \theta }\right|}^{2}},\;\mathrm{where}\;\left|\Delta \theta \left(n\right)\right|<0.5\pi \\ RMSE_{tranx}=\sqrt{\frac{1}{N}\sum_{n}{\left|\Delta x\left(n\right)-\mu_{\Delta x}\right|}^{2}}\\ RMSE_{trany}=\sqrt{\frac{1}{N}\sum_{n}{\left|\Delta y\left(n\right)-\mu_{\Delta y}\right|}^{2}} \end{array}$$

where N denotes the number of experiments; Δθ, Δx, and Δy are parameters of a similarity matrix estimated using the proposed method, and $\mu_{\Delta \theta }$, $\mu_{\Delta x}$, and $\mu_{\Delta y}$ denote the average of Δθ, Δx, and Δy, respectively. The range of Δθ(n) is limited because the side-rear-view camera cannot capture the rear-view if |Δθ(n)| exceeds 0.5π. Table 2 shows the average and the RMSE of each parameter calculated from 100 repeated experiments in different environments, and Figure 16 shows the parameter, precision, and recall values calculated over 100 experiments. If the precision and recall are 1, the two images are identical. We can see that the averages of precision and recall were 0.9758 and 0.9239, and both values were close to 1. This means that the edge image of the 3D vehicle model and the calibrated image were almost identical. The RMSE of rotational similarity was less than 1°, and the RMSE values of x- and y-axes translational similarity were 4.9041 and 13.4763 px, respectively. An RMSE value close to zero indicates that the experimental results are not affected by changes in the driving environment. Since we experimented in various environments without changing the camera orientation and the edge image of the 3D vehicle model, these quantitative results verified that the proposed method could perform online-calibration in most environments with RVA.

#### 4.2.2. Experiments with Various Cameras

FOV of cameras used in ADAS depends on its purpose. For example, forward collision warning systems and parking assistance systems commonly use a narrow-angle camera and a wide-angle camera, respectively. In some cases, lens distortion may occur. In order to verify that the proposed algorithm can work in these various conditions, we experimented with three types of cameras: a 150-degree FOV camera with lens distortion, a 115-degree FOV camera with lens distortion, and a 115-degree FOV camera without lens distortion. Camera orientation was manually regulated by 5-degree per axis.

**Figure 17:**
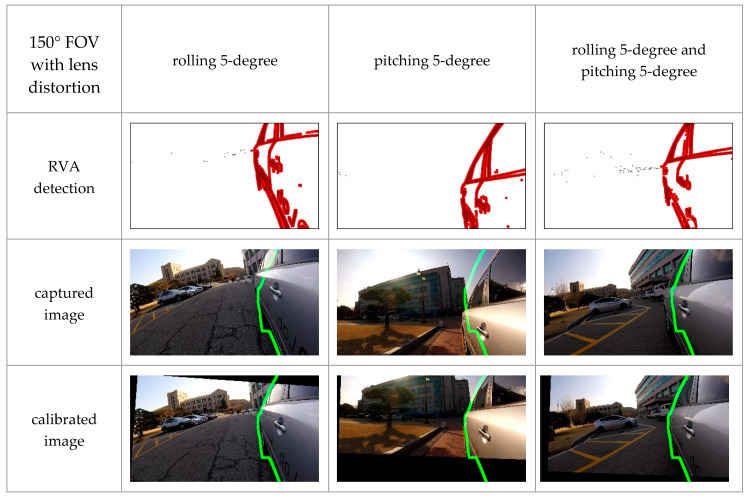
Experimental results with a 150-degree Field Of View (FOV) camera with lens distortion in different orientation conditions, where the green line denotes the boundary of the 3D vehicle model.

**Figure 18:**
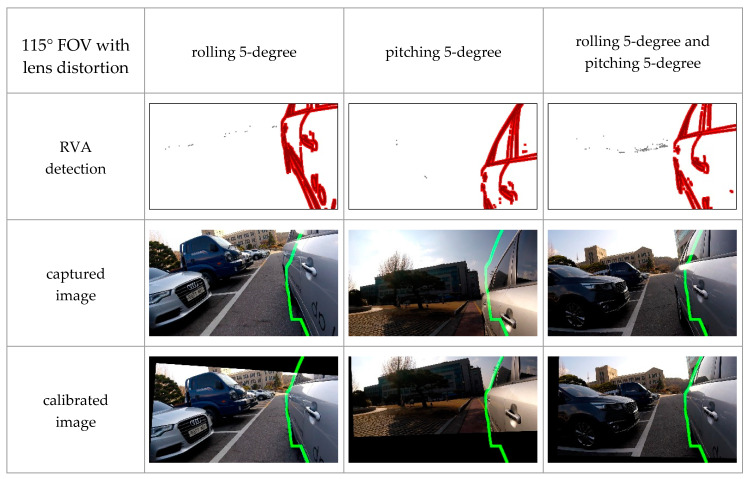
Experimental results with a 115 degree FOV camera with lens distortion in different orientation conditions, where the green line denotes the boundary of the 3D vehicle model.

**Figure 19:**
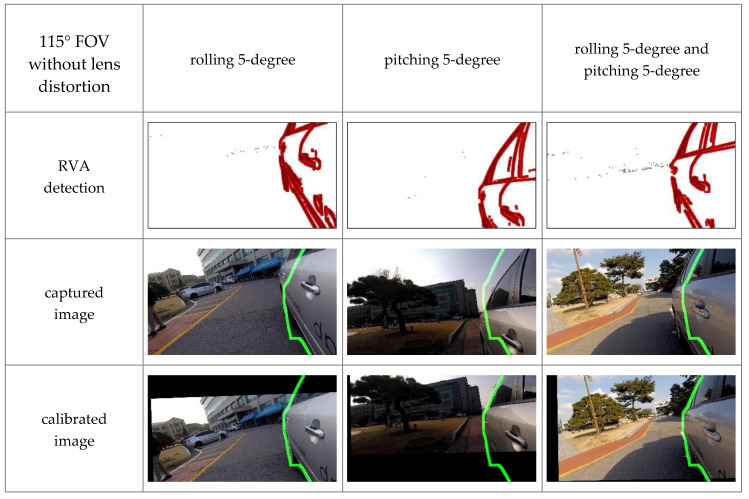
Experimental results with 115-degree FOV camera and lens distortion correction in different orientation conditions, where the green line denotes the boundary of the 3D vehicle model.

As shown in Figure 17 and Figure 18, changes in FOV did not significantly affect the experimental results. Additionally, Figure 19 shows that the proposed method could calibrate both the lens distorted-image and lens distortion corrected-image. These qualitative results indicate that the proposed method could perform online calibration even if cameras’ FOV and lens distortion were changed. Therefore, our method has the potential to be applied to various ADAS cameras.

### 4.3. Comparison with Previous Methods

As aforementioned in Section 2, camera calibration can be categorized according to which features and devices were used: offline calibration, online calibration with additional devices, and online calibration without additional devices. Table 3 shows the comparison of the related works and the proposed method from the viewpoint of the side-rear-view monitoring system calibration. Offline calibration is an inconvenient and restrictive method because the driver has to visit a large service center equipped with special facilities, and it cannot be conducted in natural driving environments. In addition, these facilities increase the price of offline calibration-based products. Likewise, additional devices of online calibration also increase the price.

Online calibration is convenient because it can automatically calibrate cameras in a natural driving situation. However, traditional online calibration can hardly calibrate side-rear-view cameras due to constraints. They must extract features such as lane from captured images, but the side-rear-view camera looking at the horizon behind the vehicle does not capture traffic lanes around the vehicle. In contrast to those methods, the proposed method can calibrate the side-rear-view camera using RVA that is being photographed at all times in natural driving environments.

Unfortunately, since there is no previous method that can calibrate side-rear-view monitoring system in natural driving environments, it is impossible to conduct quantitative performance comparison of the previous and proposed methods with the same dataset. However, the comparison summarized in Table 3 clearly explains that the proposed method is superior to the other previous methods in terms of side-rear-view camera calibration. Moreover, we can utilize the RVA information instead of the calibration patterns to implement offline calibration for calculating the similarity score and aligning images.

The similarity matrix consisting of image rotation and translation parameters can be estimated by minimizing an algebraic distance, called reprojection error, between matched feature points.

(15) $${\hat{H}}_{S}=\underset{H_{S}}{\mathrm{argmin}}\underset{i}{\sum }\|{\overset{ˇ}{m}}_{i}-H_{S}m_{i}\|{}^{2}$$

where $H_{S}$ is the similarity matrix, $m_{i}$ is i-th feature points inside the RVA in a captured image, ${\overset{ˇ}{m}}_{i}$ is i-th feature points of the 3D vehicle model corresponding to $m_{i}$, and ${\hat{H}}_{S}$ is the estimated similarity matrix. We solved Equation (15) using the Levenberg–Marquardt method, one of the maximum likelihood estimation methods [45]. Experiments were performed using three types of cameras.

**Figure 20:**
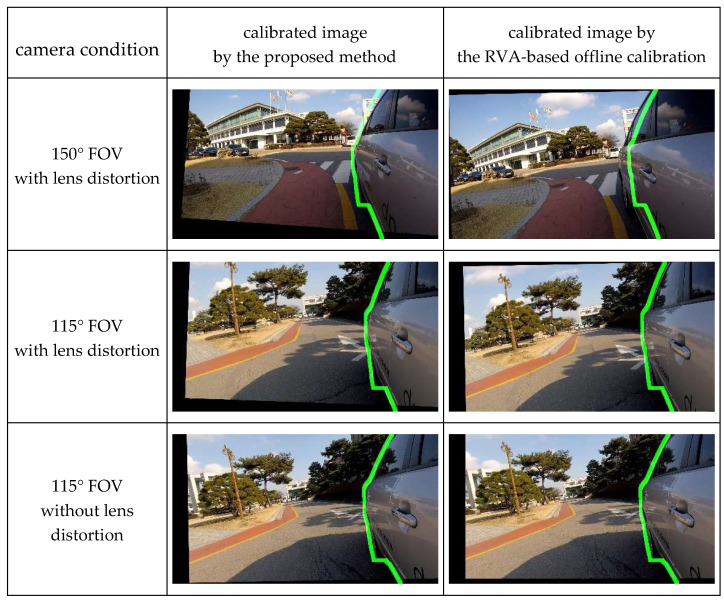
Qualitative performance comparison.

The proposed method can estimate similar parameter values to the RVA-based offline calibration method, as seen in Table 4. Furthermore, the experimental results with the 150-degree camera in Figure 20 show that the RVA boundary of the calibrated image by our method resembles the green line more than the previous methods. Additionally, other calibrated images in Figure 20 show that all RVA boundary locations of both methods almost fit the green line. These experimental results indicate that our method can provide similar results as the RVA-based offline calibration method even under conditions where the previous method cannot operate due to a lack of feature points.

### 4.4. Limitation of Calibration

**Figure 21:**
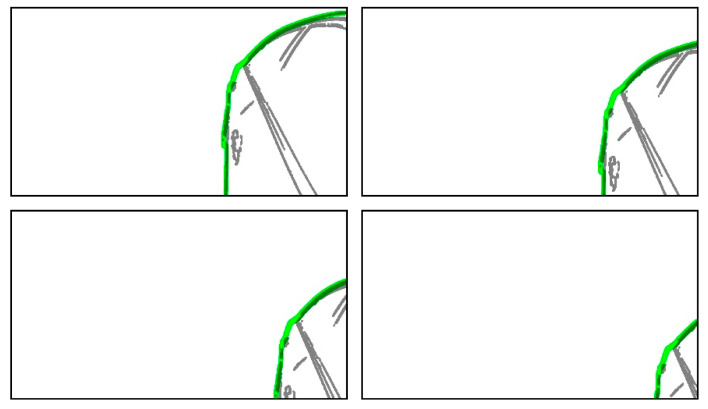
Images with gradually decreasing RVA of a 3D vehicle model image to verify the minimum required RVA, where the green lines are the boundaries of a 3D vehicle model image.

The proposed method compared the static edge points inside RVA of a captured image and a 3D vehicle model image to calculate the similarity between the two images. Therefore, the static edge points of RVA represent an essential factor, but RVA can be altered by various factors. To investigate the effect of RVA range, we repeated the experiment by gradually decreasing the RVA range. Since the goniometer has a limitation in changing the camera orientation, we decreased the RVA of the 3D vehicle model image instead of changing the camera orientation, as shown in Figure 21. We could predict that the calibrated images corresponding to Figure 21 will display the translated images along the x- and *y*-axis directions. Therefore, if the rotation parameter is changed or if the translation parameter is different from the decreased RVA value, the calibration fails.

**Figure 22:**
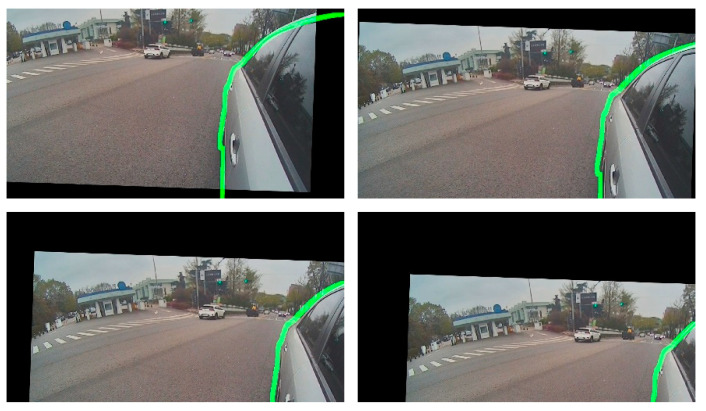
Calibrated images corresponding to Figure 21, where the green lines denote the boundaries of a 3D vehicle model image.

Figure 22 shows the calibrated images corresponding to the images in Figure 21. We can see that the rotation parameter of the bottom-right image in Figure 22 differed from those of the other calibrated images, and the vehicle boundary in all calibrated images in Figure 22 almost matched the green curves representing the boundaries of the 3D vehicle model. Accordingly, the calibration failed only in the bottom-right case. RVA in case of the failure had no static edge points inside it, unlike the other cases. This means that the calibration can fail when it uses only RVA boundary data. Through this experiment, we confirmed that the elements that serve as static edge points inside RVA (i.e., door handle or pillar) must be photographed for automatic calibration of the side-rear-view monitoring system.

## 5. Conclusions

We proposed an automatic online calibration method for the monitoring system of a vehicle equipped with a side-rear-view camera. The proposed method has the following advantages. The first advantage is that it can be used to automatically calibrate the camera while driving without using additional sensors or artificial markers. Therefore, no specialized facilities are required for calibration. In addition, there is no constraint that offline calibration must be performed before automatic calibration, which is true of conventional methods. The next advantage is that it provides consistent results, even when the driving environment changes. This is possible because we eliminate irrelevant data before utilizing RANSAC to provide consistent results in various driving environments. The third advantage is that the proposed method facilitates large-scale template matching by using information about edge points instead of color information because the method uses the Hough space. This advantage solves the problem of traditional large-scale template-matching methods that use color information, as well as the problem that the RVA color changes depending on the vehicle color and the driving environment. The last advantage is that the calibration requires only RVA information. Therefore, the proposed method can potentially be used to calibrate most cameras mounted on a vehicle.

Based on this potential, we expect the proposed automatic online calibration method to be applied not only to side-rear-view monitoring systems but also to various vision-based ADAS. These advantages indicate that the proposed method can provide convenience to motorists who require recalibration, and it can increase profits for vehicle manufacturers by reducing the usage of special facilities. As a disadvantage, the proposed method estimates the similarity instead of camera orientation. This disadvantage sometimes induces affine transformation errors. These errors can be solved by using a planar vehicle model, but it is difficult to overcome this disadvantage with the proposed method because it employs a 3D vehicle model. The results of experiments conducted in various driving environments indicate that the proposed automatic calibration method is suitable for use in real-world applications.

## Figures and Tables

**Table 1:** Definitions of true positive (TP), false positive (FP), false negative (FN), and true negative (TN) for computing precision and recall.

		$I_{m}$	
		$S_{m}$	${\left(S_{m}\right)}^{c}$
${\hat{I}}_{c}$	$S_{c}$	TP	FP
	${\left(S_{c}\right)}^{c}$	FN	TN

**Table 2:** Average and Root Mean Squared Error (RMSE) of the quantitative results of 100 repeated experiments in different environments.

	Δθ (Degree)	Δx (Pixel)	Δy (Pixel)	Precision with Calibration	Recall with Calibration	Precision without Calibration	Recall without Calibration
**Average**	−1.4000	−124.6400	−55.3000	0.9758	0.9239	0.6715	0.5929
**RMSE**	0.6164	4.9041	13.4763	-	-		

**Table 3:** Comparison of the related works and the proposed method.

Method		Driver’s Convenience	Product Cost	Calibration Constraint
Offline calibration		Poor	Poor	Fair
Online calibration with additional devices		Good	Fair	Poor
Online calibration without additional devices	Previous methods	Good	Good	Poor
	Proposed method	Good	Good	Good

**Table 4:** Quantitative performance comparison.

Camera Condition	Method	Δθ (Degree)	Δx (Pixel)	Δy (Pixel)
150° FOV with lens distortion	proposed	0	89	-25
	offline	1	75	0
115° FOV with lens distortion	proposed	−1	96	−71
	offline	1	103	−6
115° FOV without lens distortion	proposed	2	93	−33
	offline	0	117	−38
